# A Well-Trained Team During Anaphylactic Shock After Rocuronium in a Patient With Aortic Stenosis: A Case Report

**DOI:** 10.3389/fmed.2019.00305

**Published:** 2020-01-09

**Authors:** Tamar Macharadze, Andrew Davies, Igor Fedor

**Affiliations:** ^1^Cardiothoracic Critical Care Unit, Wythenshawe Hospital Manchester University, Manchester, United Kingdom; ^2^Department of Molecular Biology, Davit Tvildiani Medical University, Tbilisi, Georgia

**Keywords:** anaphylactic shock, rocuronium, aortic stenosis, cardiac surgery, cardiopulmonary resuscitation

## Abstract

A 66-year-old patient with aortic stenosis was scheduled for an aortic valve replacement and coronary artery bypass surgery. Anesthesia was induced by intravenous injection of midazolam, fentanyl, and propofol. After administration of rocuronium, he developed anaphylactic shock, which was diagnosed by clinical signs, vital parameters, and unresponsiveness to the usual vasopressors. After 30 min of cardiopulmonary resuscitation, the patient survived without any neurological deficits. This case is a reminder that early recognition and treatment of intraoperative hypersensitivity reactions are imperative. Anesthetists should also receive simulation training to achieve an adequate experience in a safe environment. With a well-trained team, it is possible to save the life of patients with aortic stenosis.

## Background

Anaphylaxis is a life-threatening, critical event that often happens suddenly and can be fatal (1). The incidence of perioperative anaphylaxis varies between 1:6,000 and 1:20,000 anesthetics (2). According to the sixth National Audit Project of the Royal College of Anesthetists (NAP6), muscle relaxants are second only to antibiotics as a trigger of anaphylaxis perioperatively (3).

We describe for the first time an anaphylactic shock caused by rocuronium in a patient with an aortic stenosis (peak gradient 60 mmHg, mean gradient 30 mmHg). The intraoperative hypersensitivity diagnosis is difficult to diagnose, as the symptoms are similar to the anesthesia effects on the cardiovascular and respiratory systems. That is why it has been suggested that anaphylaxis should be considered in all cases where hypotension is not responding to the usual vasopressors (4). Here, we would like to underline how important an early recognition of anaphylactic shock is in patients and what a big role it plays for anesthetists to have an appropriate training of management, because this is a rare event. In the literature, there are several case reports about the anaphylactic shock to rocuronium (5), but we describe it for the first time in a cardiac patient with aortic stenosis who survived without a neurological deficits after a resuscitation. In the current report, we will show that the life of a patient can be saved even with such a severe disease.

## Case Presentation

A 66-year-old, 96-kg, 177-cm American Society of Anesthesiologists Classification (ASA) III male without history of general anesthesia, with hypertension (bisoprolol 5 mg, amlodipine 10 mg, and olmesartan 40 mg) aortic stenosis and hypercholesterolemia, was admitted to our hospital complaining of a recent onset of angina pectoris. He remained symptomatic at that time. On cardiac auscultation, he had an ejection systolic murmur at the apex, consistent with aortic stenosis, which radiated into both carotid arteries. His blood pressure was 150/65 mmHg. Carotid duplex identified mixed and dense plaques in the right and left internal carotid arteries, causing less than 50% and less than 40% stenosis, respectively. Echocardiogram revealed moderate aortic stenosis and good left ventricular (LV) function. Dobutamine stress echocardiogram demonstrated significant left anterior descending territory ischemia, which was been confirmed to be due to left anterior descending coronary artery (LAD) stenosis on coronary angiography. The patient was scheduled for an aortic valve replacement (AVR) and coronary artery bypass graft (CABG) × 1 surgery.

On the day of surgery, a radial arterial line was inserted using 1 ml of lidocaine while in the operating room and was used for the blood pressure measurement. Anesthesia was induced through a peripherally inserted 16G cannula with midazolam 3 mg, fentanyl 500 μg, and propofol 100 mg. The blood pressure immediately dropped, necessitating metaraminol 0.5 mg intravenously, which raised it to 150/90 mmHg. Shortly after the injection of rocuronium 100 mg, the patient developed unrecordable hypotension 40/10 mmHg needing cardiopulmonary resuscitation (CPR), which caused the heart rate to increase from 70 to 150 bpm. He had severe bronchospasm, and mask ventilation was difficult. There was red flushing of the skin, cyanosis, and desaturation (SpO_2_ 73%). The patient did not respond to a further dose of metaraminol 5 mg. At this time, anaphylaxis was diagnosed. The patient required tracheal intubation, and fluid resuscitation (crystalloids 3,000 ml, two units of red blood cells, and 5% albumin 1,000 ml) was started. There was no response on epinephrine 100 μg and 1 mg of boluses. Because we did not know the cause of the allergic reaction, we administered sugammadex 1,600 mg and 30 ml of bolus of 20% intralipid solution over a period of 1 min. The heart rate dropped to 80 bpm, but despite ongoing CPR, the blood pressure remained at 60/40 mmHg. There was still no response to a further epinephrine 1 mg of bolus, and the cardiac surgeons started to prepare the right common femoral artery and right femoral vein for a peripheral extracorporeal membrane oxygenation (ECMO) circuit to allow CPR to continue, which was considered after the cardiac arrest. A central venous catheter was inserted in the left internal jugular vein of the patient. At this point, after 32 min of resuscitation, the blood pressure increased to 100/50 mmHg, CPR was stopped, and as there was no longer a need for ECMO, the groin wound was closed. We started a vasopressin infusion of 5.2083 × 10^−4^ mg/kg/min, epinephrine infusion of 3.47 × 10^−5^ mg/kg/min, and norepinephrine infusion of 1.8519 × 10^−5^ mg/kg/min. Hemodynamic stability was achieved. We excluded pneumothorax with percussion as it was resonant and equal on both sides. Afterwards, we did an emergency bronchoscopy to clear all the major airways because ventilation was still difficult, with no air entry into the right lung and wheezing in the left lung. Thick white secretions were aspirated. We gave nebulizers (ipratropium bromide 0.5 mg and salbutamol sulfate 2.5 mg), via the endotracheal tube, which had an immediate effect. After the acid-base balance and plasma glucose levels were stabilized, the patient gradually recovered and was transferred to cardiac intensive care unit for further treatment and monitoring. It was not assumed safe to proceed with surgery on this admission, and the patient was referred urgently to the immunology service. Twenty hours after the cardiac arrest, he was extubated, and 1 day after extubation, he was discharged home fully orientated and without any neurological deficits.

Six weeks later, skin tests (skin prick and intradermal) were performed with histamine as a positive control and saline as a negative control against opioids, local anesthetics, and neuromuscular blocking drugs. The skin prick test revealed a positive reaction to rocuronium. An intradermal test showed a positive reaction to vecuronium and pancuronium. After the prick test result, we avoided performing an intradermal test of rocuronium. On the basis of these results, we concluded that the rocuronium was the causative agent of the anaphylactic reaction during the induction of general anesthesia.

Later, the patient returned to the hospital for surgery when anesthesia was performed without using any neuromuscular blocking drugs.

## Discussion

Most studies have identified neuromuscular blocking agents (NMBAs) as the commonest causes of anaphylaxis during general anesthesia (6), although the NAP6 suggested that antibiotics are now the commonest trigger of anaphylaxis (3). Among NMBAs, rocuronium seems to be in second place after succinylcholine as the culprit agent (3). Rocuronium-induced anaphylaxis is multifactorial, although it is thought that one of the causes is well-known allergic potential quaternary ammonium ion, which also appears in many environmental chemicals like drugs, disinfectants, toothpaste, and shampoo (7). It has been shown that allergy toward NMBAs can develop after ingestion of antitussive syrup containing pholcodine, because it stimulates asymptomatic production of antibodies (8, 9) and increases IgE levels in sensitized individuals (10). It has been shown that NMBA sensitization has decreased in Norway since the withdrawal of pholcodine medicine (11). It is difficult to say, but the patient may well have been sensitized by environmental substances or pholcodine. It has to be mentioned that patient was not taking any angiotensin-converting enzyme inhibitor, he had no history of medicament allergy, and he was going under general anesthesia for the first time in his life.

Anaphylaxis needs immediate management (12) such as removing the triggering agent, securing the airways, stabilizing circulation, administering sympathomimetics, and supplementing vascular fluid. Prompt recognition and treatment of anaphylaxis are imperative, but health-care professionals often fail to recognize and diagnose early signs and symptoms of the condition. This was pertinent in this patient who, owing to his aortic stenosis, was at risk of a severe decrease in blood pressure on induction of anesthesia. There could well have been a delay in diagnosing anaphylaxis. Anesthetists commonly have a tendency to overlook allergy as the cause of circulatory/ventilatory collapse during induction. As we mentioned above, clinical signs, vital parameters, and non-response to the usual vasopressors are key points in diagnosing anaphylactic shock. In our case, the team members underwent hands-on training practice that was organized by the hospital simulation center.

As after the cardiac arrest it became incredibly difficult to maintain blood pressure in spite of doing CPR and giving boluses of epinephrine, we considered putting the patient on peripheral venoarterial (VA) ECMO from the very beginning. There is always an ECMO-skilled surgeon on call, and most of the anesthetists are proficient in percutaneous venous and arterial cannulation in our hospital. During ongoing CPR, groins were already dissected for cannula insertion, but a return of spontaneous circulation (ROSC) occurred, so there was no need to support the patient by ECMO.

## Conclusion

In our patient, anaphylactic shock occurred during induction of anesthesia. We subsequently found that the cause was rocuronium, a non-depolarizing muscle relaxant. Improving awareness of perioperative anaphylaxis among anesthetists promotes quick diagnosis and adequate medical treatment. Simulation training in intraoperative hypersensitivity reactions (13) and hands-on practice for resuscitation (14) are ways to achieve adequate experience in a safe environment. When the team is well-trained and effective, it is possible to save the life even of patients with aortic stenosis.

## Ethics Statement

All clinical data in this case report were collected with the consent of the patient. Written informed consent was obtained from the patient for the publication of this report.

## Author Contributions

TM provided intraoperative care and designed and wrote the manuscript. AD provided intraoperative care and was the physician of record. IF provided intraoperative care and edited the manuscript.

### Conflict of Interest

The authors declare that the research was conducted in the absence of any commercial or financial relationships that could be construed as a potential conflict of interest.

## Abbreviations

LV: left ventricular
LAD: Left anterior descending coronary artery
AVR: aortic valve replacement
CABG: coronary artery bypass graft
ECMO: extracorporeal membrane oxygenation circuit
NMBA: neuromuscular blocking agents.
